# AI-, VR-, and Exergame-Based Dance and Movement Research on Psychological Outcomes: A Bibliometric and Topic-Modeling Analysis of Thematic Structure and Development

**DOI:** 10.3390/healthcare14121662

**Published:** 2026-06-11

**Authors:** Mingzhu Wu, Hongfei Zhang, Kunpeng Li, Mariusz Lipowski, Wenjun Hu

**Affiliations:** 1 Gdansk University of Physical Education and Sport, 80-336 Gdansk, Poland; mingzhuwu4113sport@163.com (M.W.); zhanghongfei77@126.com (H.Z.); tsunami900@163.com (K.L.); 2 Social and Humanities Department, WSB Merito University in Gdansk, 80-226 Gdansk, Poland; mariusz.lipowski@gdansk.merito.pl

**Keywords:** artificial intelligence, virtual reality, exergaming, dance and movement, psychological outcomes, topic modeling

## Abstract

Artificial intelligence (AI), virtual reality (VR), and exergame technologies have been increasingly used in dance and movement activities. However, this literature remains dispersed across different areas, making it difficult to determine how the field has developed. This study mapped the research landscape and thematic development of AI-, VR-, and exergame-based dance and movement research on psychological outcomes using bibliometric analysis and latent Dirichlet allocation (LDA) topic modeling. A total of 252 records indexed in the Web of Science Core Collection from 2011 to 2025 were included. Five related thematic strands were identified: immersive dance interaction and technology-supported teaching; rehabilitation-oriented dance or rhythm training; school-based exergaming and psychophysiological assessment; behavioral program design and intervention implementation; and AI-based motion or emotion recognition. These strands indicate that the field has developed into a multi-layered research space shaped by technology functions, movement contexts, intervention formats, and psychological constructs, rather than a single dance-intervention or technology-application domain. At the same time, psychological outcomes were not represented with equal clarity across these strands. Participation-related and psychosocial constructs, including enjoyment, motivation, engagement, self-efficacy, social interaction, emotional expression, and quality of life, were more frequently represented, whereas mental-health-related outcomes such as anxiety, depression, stress, loneliness, and psychological well-being were less consistently connected to technology-supported dance or movement interventions. These findings clarify where evidence is concentrated, how major themes are organized, and where psychological outcome measurement requires clearer theoretical and methodological specification. Future studies should use comparative and longitudinal designs to examine whether VR/AI-based feedback-supported movement training offers added value over conventional dance or movement programs for psychological outcomes, participation, exercise experience, and longer-term behavior change.

## 1. Introduction

Regular physical activity is widely recognized as an important contributor to physical and psychological health. Within this broader field, dance and dance-like activities occupy a distinctive position because they combine physical exertion, rhythm, music, bodily expression, and social interaction. These features make dance-based activities more than a form of exercise: they may also create conditions for enjoyment, emotional expression, self-perception, and social connection. As a result, dance-based programs have been increasingly examined in educational, community, and health-related contexts as movement activities with potential psychological relevance [1,2,3].

Recent advances in artificial intelligence (AI), virtual reality (VR), augmented reality (AR), mixed reality (MR), and exergaming have expanded the ways in which dance and movement-based activities can be designed, delivered, and evaluated. VR and exergame systems can provide immersive environments, interactive tasks, real-time feedback, and structured training scenarios, while AI methods can be used to recognize movement patterns, evaluate performance, classify affective expression, and support automated feedback. These developments suggest new possibilities for linking dance-based activity with psychological outcomes, but they also make the literature more difficult to organize because similar forms of work may be described using different technological and disciplinary terms [4,5,6,7].

A further challenge concerns the breadth of psychological outcomes examined in this literature. Studies on dance, exergaming, VR training, rehabilitation, and AI-based movement analysis often assess constructs that differ substantially in theoretical meaning and measurement practice. Some outcomes capture immediate motivational or experiential responses, such as enjoyment, motivation, engagement, flow, and perceived competence. Others reflect psychosocial functioning, including self-efficacy, self-esteem, body image, social connectedness, social interaction, inclusion, and emotional expression. A third group concerns mental health and well-being, including anxiety, depression, stress, quality of life, and psychological well-being. Distinguishing these levels is necessary for interpreting whether the mapped literature primarily addresses participation-related experiences, broader psychosocial functioning, or more direct indicators of mental health and well-being. In this study, these categories were used as a conceptual guide for interpreting keyword patterns and LDA-derived topics, rather than as fixed outcome groups for effect-size synthesis.

Existing studies are dispersed across partially overlapping areas, including physical education, community-based activity promotion, rehabilitation, exergaming, immersive training, and AI-based movement or affect recognition. However, the relationships among these areas remain insufficiently clarified. In particular, it is not yet clear where research activity is concentrated, how AI-, VR-, and exergame-based technologies are being used, which psychological outcomes are most frequently addressed, and whether technology-oriented studies are connected to intervention-oriented evaluation in real-world settings. These issues are difficult to answer from isolated reviews focused on a single population, intervention type, technology, or outcome category [8,9,10,11].

Previous reviews have provided valuable syntheses of specific segments of this field, such as exergames and active video games, VR-based rehabilitation, dance/movement therapy, and AI-based movement recognition. These reviews are useful when the purpose is to assess intervention effects in a defined population or to summarize evidence for a specific technology. However, the broader literature on AI-, VR-, and exergame-based dance and movement research remains dispersed across educational, community-based, rehabilitation, and computational contexts. The function of technology also differs across studies: VR and exergaming are commonly used as intervention or training platforms, whereas AI is often applied to movement recognition, emotion classification, automated evaluation, and model development. Psychological outcomes are also reported in different ways and are not always treated as primary or clearly defined targets. Therefore, before more targeted effectiveness questions can be examined, it is necessary to map how these intelligent technologies have been applied and developed in dance and movement research, what thematic patterns have emerged, and how psychological outcomes are represented. A bibliometric and LDA topic-modeling approach is suitable for this purpose because it can describe publication patterns, keyword relationships, intellectual structures, latent themes, and temporal development across a dispersed interdisciplinary literature [12,13,14,15].

Accordingly, the present study uses bibliometric analysis and LDA-based topic modeling to examine AI, VR, and exergame-based dance and movement research on psychological outcomes from 2011 to 2025. The study aims to: (1) describe the structural development of the field; (2) identify the main thematic patterns linking dance/movement activities, AI/VR/exergame technologies, and psychological outcomes; (3) examine how different types of psychological outcomes are represented across the literature; and (4) clarify how research priorities have evolved over time. By doing so, this study seeks to provide a more organized account of an emerging interdisciplinary area and to identify directions for linking AI/VR-supported dance and movement research with clearer psychological targets and validated outcome assessment.

## 2. Materials and Methods

### 2.1. Search Strategy and Data Retrieval

**Figure 1:**
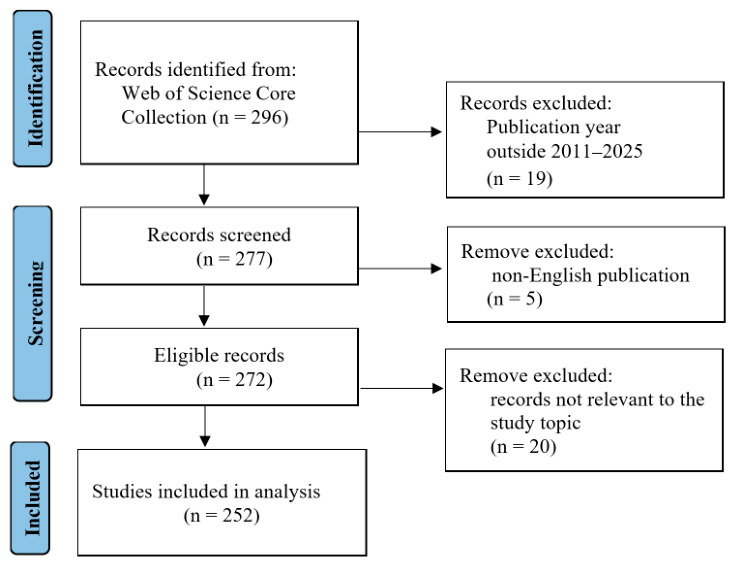
Flowchart of literature screening and study selection.

The literature search was conducted in the Web of Science Core Collection (WoSCC) using the advanced search interface. WoSCC was selected to maintain consistency between the bibliometric analysis and the LDA topic-modeling analysis. The present study required a unified source of standardized bibliographic records, citation data, author information, institutional affiliations, keywords, abstracts, and cited references so that publication trends, keyword co-occurrence, co-citation patterns, collaboration networks, and topic-modeling results could be generated from the same corpus. Therefore, the interpretation of the results is limited to the WoSCC-indexed corpus analyzed in this study. The search strategy was constructed around three concept clusters: (1) AI-, immersive-, and game-based technologies; (2) dance, dance sport, rhythm-based, and dance-like movement activities; and (3) psychological, psychosocial, and mental health-related outcomes. The search covered the period from January 2011 to December 2025 and was conducted in the Topic field. Only records indexed in WoSCC at the time of retrieval were included, including early-access or online-first records where applicable. The third cluster was intentionally broad because psychological outcomes in this field are reported using diverse terms, ranging from motivational and experiential constructs to psychosocial and mental-health-related constructs. To reduce the risk of retrieving studies in which these terms were only peripheral, the search required the simultaneous presence of all three concept clusters, and the retrieved records were then screened for relevance to the study focus. The following Boolean search string was used: (“virtual reality” OR VR OR “augmented reality” OR AR OR “mixed reality” OR MR OR “extended reality” OR XR OR exergame OR “active video game” OR “interactive video game” OR “serious game” OR “artificial intelligence” OR “machine learning” OR “deep learning”) AND (“dance sport” OR “sports dance” OR “dance-based exercise” OR “dance based exercise” OR “dance game “ OR “rhythm game” OR “rhythm-based exercise” OR “dance/movement therapy” OR “dance movement therapy” OR dance OR ballroom) AND (psychology OR “mental health” OR emotion OR affect OR mood OR anxiety OR depression OR stress OR “self-efficacy” OR “self efficacy” OR “self-esteem” OR “self esteem” OR motivation OR enjoyment OR engagement OR “flow experience” OR “flow state” OR “body image” OR “well-being” OR wellbeing OR “quality of life” OR “QoL” OR “social connectedness” OR “social connection” OR “social interaction” OR loneliness OR empathy). The initial query retrieved 296 records. After excluding records outside the 2011–2025 period (*n* = 19), non-English publications (*n* = 5), and records not relevant to the study focus (*n* = 20), 252 records were retained and exported in plain-text format with full bibliographic information and cited references for subsequent bibliometric and topic-modeling analyses. The screening and eligibility process is summarized in Figure 1. To improve screening reliability, the excluded records and uncertain cases were checked by a second author during revision, and any disagreements were resolved through discussion.

### 2.2. Eligibility Criteria

All retrieved records were screened according to predefined eligibility criteria. Records were included if they: (1) were published between January 2011 and December 2025; (2) were written in English; (3) addressed AI-, VR-, AR-, MR-, XR-, exergame-, or machine-learning-related technologies; (4) involved dance, rhythm-based, dance-like, or movement-based activities; and (5) included psychological, psychosocial, affective, motivational, or mental-health-related outcomes or constructs. Records were excluded if they were outside the time window, were not written in English, did not involve dance or dance-like movement activities, did not include a relevant technology component, or mentioned psychological terms only peripherally without relevance to the study focus.

Two authors were involved in the screening process. The first author conducted the initial screening of titles, abstracts, and, where necessary, full texts according to the predefined eligibility criteria. A second author then checked the excluded records and uncertain cases to reduce the risk of inappropriate exclusion or misclassification. Any disagreements were resolved through discussion.

### 2.3. Data Analyses

The analysis combined bibliometric visualization and LDA-based topic modeling. Bibliometric analysis was used to describe publication trends, keyword relationships, collaboration networks, and co-citation patterns. LDA topic modeling was used to identify latent themes in titles and abstracts and to examine how these themes changed over time.

The 252 eligible records were imported into CiteSpace (Version 6.3.R1; Drexel University, Philadelphia, PA, USA) for bibliometric visualization and network analysis. CiteSpace was used for keyword co-occurrence, co-citation analysis, burst detection, betweenness centrality calculation, LLR-based cluster labeling, and time-zone visualization. The time span was set from 2011 to 2025, with one year per time slice. The g-index was used as the selection criterion, with k = 25, and minimum spanning tree pruning was applied. Networks were constructed for keywords, countries/regions, institutions, authors, cited references, and cited journals. Node size represented frequency, and betweenness centrality was used to identify bridging nodes. Keyword clusters were labeled using the log-likelihood ratio algorithm. The keyword clustering solution showed a clear modular structure and good internal consistency, with modularity Q = 0.61 and weighted mean silhouette S = 0.8888. VOSviewer (Version 1.6.17; Centre for Science and Technology Studies, Leiden University, Leiden, the Netherlands) was used as a complementary visualization tool to check network layouts and support the interpretation of co-occurrence and collaboration structures.

LDA topic modeling was performed on the titles and abstracts of the same 252 records. Before LDA modeling, the titles and abstracts were combined as the text source for each record and subjected to standard text cleaning. The text was converted to lowercase, and punctuation, numbers, non-informative symbols, and common English stop words were removed. General academic terms with limited thematic meaning, such as “study”, “result”, “method”, “paper”, and “analysis”, were also removed where appropriate to reduce noise. Multi-word expressions that were central to the research topic, such as “virtual reality”, “active video game”, “quality of life”, “deep learning”, and “machine learning”, were checked during topic interpretation to avoid misinterpreting domain-specific phrases as unrelated single words. The cleaned document–term matrix was then used for LDA estimation. Candidate topic solutions from K = 2 to K = 9 were compared using topic coherence (C_v) and perplexity. Coherence was used to assess the semantic interpretability of topics, while perplexity was used as an indicator of model fit. The K = 5 solution produced the highest coherence value among the tested solutions, indicating the best semantic interpretability within this range. Although perplexity continued to decrease as K increased, larger K solutions divided closely related terms into smaller and less coherent thematic fragments when the high-probability terms and representative documents were inspected. Therefore, K = 5 was retained because it provided the most reasonable balance between semantic interpretability, model fit, and parsimony for descriptive topic mapping. This choice was treated as an interpretive modeling decision rather than as a statistically definitive topic number.

## 3. Results

### 3.1. Analysis of Theme Evolution and Frontiers in AI-Driven Decision Support

This study analyzes artificial intelligence and virtual reality in dance sport for psychological health using CiteSpace and standard bibliometric techniques, complemented by LDA topic modeling. The results are reported in four parts. First, annual publication trends are presented to describe the overall growth of the field. Second, research themes and their evolution over time are examined through keyword co-occurrence networks, cluster analysis, timeline and time-zone views, burst detection, and LDA-derived topics. Third, collaboration patterns are characterized using co-authorship networks at the country or region, institution, and author levels, with attention to prominent hubs and collaboration clusters. Fourth, the intellectual base and journal-level linkages are summarized using journal and document co-citation networks together with a journal dual-map overlay, providing an overview of the core knowledge structure in AI- and VR-supported dance sport interventions for psychological health.

#### 3.1.1. Analysis of Publication Trends

**Figure 2:**
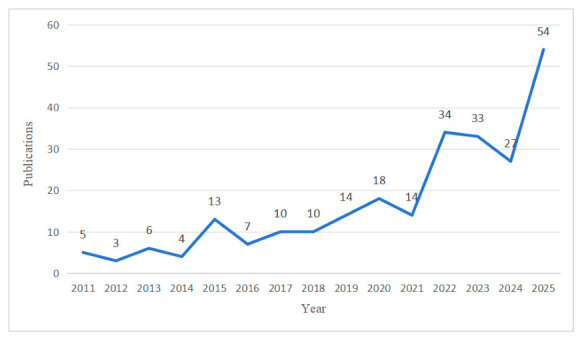
Trends in the annual number of publications from 2011 to 2025.

Annual publication output showed an overall upward pattern from 2011 to 2025 (Figure 2). From 2011 to 2015, output remained below 15 papers per year; from 2016 to 2021, it fluctuated between 7 and 18 papers per year; and from 2022 onward, it rose above 25 papers per year, reaching 54 publications in 2025. This pattern suggests growing scholarly attention to AI-, VR-, and exergame-based dance and movement research on psychological outcomes.

#### 3.1.2. Keyword Analysis

**Figure 3:**
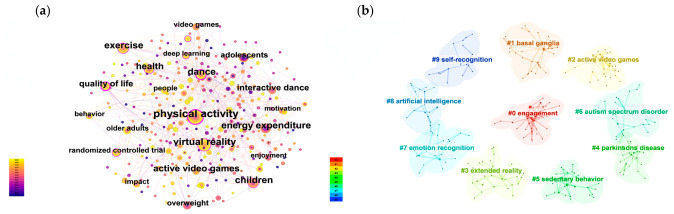
Keyword Analysis. (**a**) Construction of Keyword Co-occurrence Network; (**b**) Topic Clustering Features and Cluster Label Extraction. Larger nodes indicate higher keyword frequency, links indicate co-occurrence relationships, and colors are used to distinguish clusters or network attributes in the visualization.

Keyword co-occurrence analysis identified 333 distinct keywords and formed a dense network of terms related to dance and movement activities, VR/exergaming, physical activity, and psychological or health-related outcomes (Figure 3a). The ten most frequent keywords were physical activity (46 occurrences), virtual reality (32), exercise (28), energy expenditure (27), children (26), dance (26), health (24), active video games (24), quality of life (20) and interactive dance (19). Among these, quality of life (betweenness centrality = 0.28), dance (0.26), physical activity (0.23), virtual reality (0.17), health (0.14), randomized controlled trial (0.12), and deep learning (0.10) showed centrality values at or above 0.10. In CiteSpace-based bibliometric interpretation, nodes with betweenness centrality values of 0.10 or higher are generally interpreted as having a potential bridging role in the network. Therefore, these keywords were interpreted as potential bridging terms within the present keyword network. This threshold was used as an interpretive criterion rather than as a statistical significance threshold.

Cluster analysis of the keyword network yielded ten clusters with good internal consistency (modularity Q = 0.61; weighted mean silhouette S = 0.8888) (Figure 3b). The largest cluster #0 is labeled engagement and is characterized by terms such as physical activity, adolescents, obesity, children, sedentary behavior and active video games. Cluster #2 (active video games) focuses on game-based interventions, moderate-to-vigorous physical activity, energy expenditure and pediatric obesity. Cluster #3 (extended reality) combines extended reality, virtual reality, mental health, artificial intelligence and dance movement therapy. Other prominent clusters include basal ganglia and Parkinson’s disease (#1 and #4), sedentary behavior in young adults and college students (#5), autism spectrum disorder and fundamental movement skills (#6), emotion recognition and deep learning (#7), applications of artificial intelligence in ageing and dementia (#8), and self-recognition and body representation (#9).

**Figure 4:**
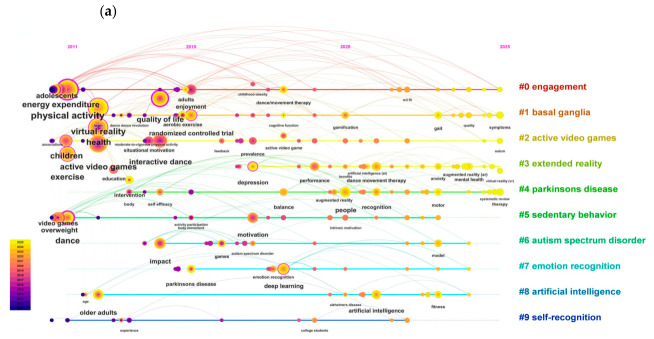

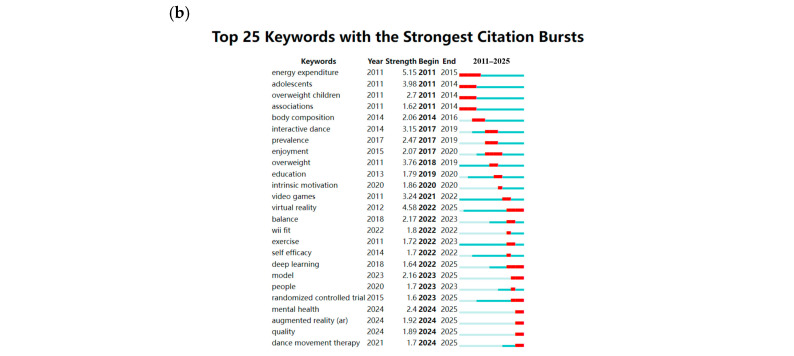
(**a**) The evolution trajectory of themes under the time zone map; (**b**) Keywords with the Strongest Citation Bursts. Node size represents keyword frequency, links indicate co-occurrence relationships, and red bars indicate the active burst period.

As shown in the timeline view and burst-detection results (Figure 4), the focus of AI- and VR-related dance and dance-like interventions has shifted over time. In the early years (around 2011–2016), burst keywords such as energy expenditure, adolescents, overweight children, body composition, exercise and video games were prominent, consistent with Cluster #0 (engagement) and Cluster #2 (active video games), and mainly related to exergames and physical-activity promotion in children and youth. Between approximately 2017 and 2020, new bursts appeared for interactive dance, prevalence, enjoyment, education and intrinsic motivation, and clusters on sedentary behavior (#5) and autism spectrum disorder (#6) became more active, indicating increased attention to motivational and educational aspects, sedentary lifestyles and special populations. From 2021 onward, strong bursts were observed for video games, virtual reality, balance, self-efficacy, deep learning, model, randomized controlled trial, mental health, augmented reality (AR), quality and dance movement therapy. These recent bursts align with clusters such as extended reality (#3), emotion recognition (#7), artificial intelligence (#8) and self-recognition (#9), and reflect a growing emphasis on immersive VR/AR environments, AI-based analysis, psychological health outcomes and more rigorous trial designs.

#### 3.1.3. Country and Institution Analysis

**Figure 5:**
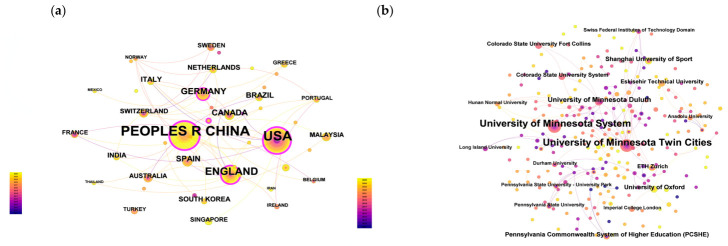
(**a**) Map of countries; (**b**) Institutional collaboration network. Larger nodes indicate higher publication frequency, links indicate collaboration relationships, and colors are used to distinguish temporal or network attributes generated by the visualization software.

In the country co-authorship network (Figure 5a), the People’s Republic of China and the USA were the most frequently represented countries in the WoSCC-indexed corpus. China has the highest publication frequency (75) and a betweenness centrality of 0.43, and the USA shows a similar pattern with 67 publications and the highest centrality value (0.44). England constitutes the next tier, with 24 publications and a centrality of 0.25, followed by Germany (13, 0.14) and Canada (11, 0.06). Several other countries contribute a moderate number of papers, including Spain (10), Brazil (9), Italy (8), India, South Korea and the Netherlands (all 7), as well as Australia, Malaysia, Switzerland, France, Japan, Sweden and Singapore (5–6 publications). Switzerland (0.10) and France (0.08) showed comparatively higher centrality values than some countries with similar publication output, indicating their bridging positions within the country co-authorship network.

In the institutional co-authorship network (Figure 5b), the University of Minnesota group was the most frequently represented institutional contributor. The University of Minnesota Twin Cities and the University of Minnesota System each appear 16 times, and the University of Minnesota Duluth appears 10 times in the network. A second tier of institutions includes Shanghai University of Sport, the Pennsylvania Commonwealth System of Higher Education, the University of Oxford and Eskisehir Technical University (all 5 publications), followed by Colorado State University System, Colorado State University Fort Collins and ETH Zurich (3 publications each). Most institutional betweenness centrality values were low (0–0.02), suggesting that institutional collaboration in this corpus was relatively dispersed rather than organized around a single dominant institutional hub.

#### 3.1.4. Document and Journal Analysis

**Figure 6:**
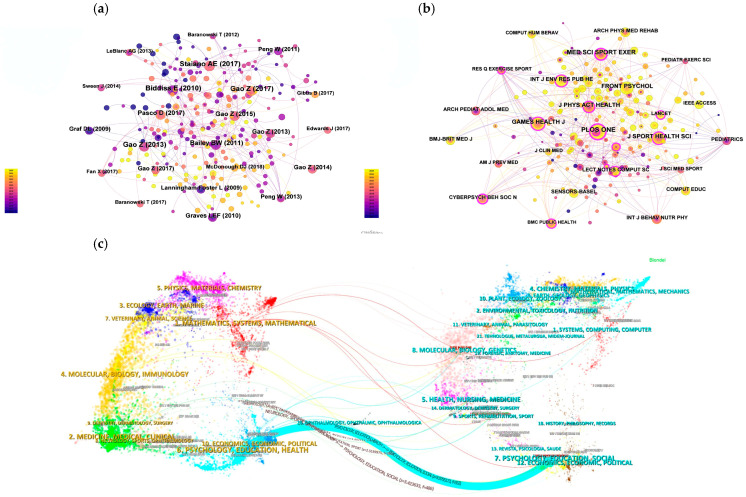
(**a**) Document co-citation network; (**b**) Journal co-citation network; (**c**) The dual-map overlay of citing and cited journals. In the co-citation networks, larger nodes indicate higher co-citation frequency, links indicate co-citation relationships, and colors/dots are used to distinguish temporal or network attributes generated by the visualization software.

The document co-citation analysis (Figure 6a, Table 1) highlights a small set of references at the core of the literature. Biddiss and Irwin (2010, *Archives of Pediatrics & Adolescent Medicine*) is the most frequently co-cited paper (12 co-citations). A series of studies by Gao and colleagues—including articles in the *Journal of Sport and Health Science* (2013, 2017) and *Obesity Reviews* (2015)—also occupy central positions, together with work by Staiano (2017), Bailey (2011), Pasco (2017), Graves (2010) and Peng (2013). These papers form the main empirical base on exergames, pediatric obesity and youth physical activity, which later studies on game-based and dance-like interventions repeatedly reference.

Figure 6b presents the journal co-citation network. The core knowledge sources are concentrated in sport and physical-activity, pediatrics, public-health and psychology outlets. *PLOS ONE* has the highest co-citation frequency, followed by *Medicine & Science in Sports & Exercise*, *Games for Health Journal*, *Journal of Physical Activity and Health*, *Frontiers in Psychology* and *Journal of Sport and Health Science*. Frequently co-cited journals such as *International Journal of Environmental Research and Public Health*, *International Journal of Behavioral Nutrition and Physical Activity* and *Archives of Pediatrics & Adolescent Medicine* further reflect the contribution of clinical and population-health research to this field.

The journal dual-map overlay in Figure 6c clarifies the disciplinary configuration. Citing journals cluster mainly in areas labeled Medicine/Medical/Clinical, Health/Nursing/Medicine, Psychology/Education/Health and Sports/Rehabilitation, with additional contributions from Systems/Computing/Computer. On the cited side, the dominant knowledge bases lie in Health/Nursing/Medicine, Psychology/Education/Social and Systems/Computing/Computer. Overall, studies on AI- and VR-supported dance and dance-like interventions for psychological health rest on an interdisciplinary foundation linking health sciences, sport sciences and computer-related fields.

### 3.2. LDA-Based Topic Modeling and Evolutionary Trend Analysis

**Figure 7:**
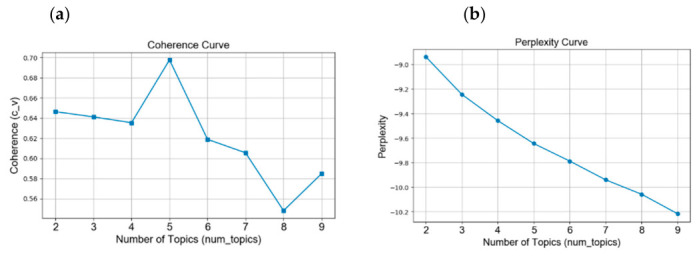
Perplexity and Coherence Evaluation. (**a**) Coherence C_v vs. Number of Topics; (**b**) Perplexity vs. Number of Topics.

To characterize latent thematic structures in the corpus, Latent Dirichlet Allocation (LDA) models were estimated on the titles and abstracts of the 252 records. Candidate solutions with 2–9 topics (K = 2–9) were compared using topic coherence (C_v) and model perplexity (Figure 7). On the basis of these indices, a five-topic solution (K = 5) was retained and used for the subsequent description of topic content and temporal evolution, with the top terms and weights for each topic provided in Table S1.

#### 3.2.1. Perplexity and Coherence Evaluation

As shown in Figure 7, the coherence curve increases from K = 2 and reaches its maximum at K = 5 (C_v ≈0.70), then declines as additional topics are added. Perplexity decreases monotonically with increasing K and attains its lowest value at K = 9, while values from K = 5 onward are already relatively low. Considering the highest coherence value at K = 5, the inspection of high-probability terms and representative documents, and the need for a parsimonious structure for descriptive mapping, K = 5 was selected for subsequent thematic description. Because perplexity continued to decrease for larger K values, this choice was interpreted as a balance between model fit and topic interpretability, rather than as a statistically definitive topic number.

#### 3.2.2. Topic Overview and Analysis

Based on the selected K = 5 solution, high-probability terms and document–topic distributions were examined to interpret each topic. The five LDA topics should be understood as probabilistic thematic patterns within the retrieved corpus rather than as fixed categories of intervention studies. Because the corpus was defined by the intersection of technology-related terms, dance/rhythm-based movement terms, and psychological or mental-health-related terms, the topics differ in how these elements are combined. Some topics emphasize VR or exergame-based intervention and training platforms, whereas others emphasize AI-based recognition, automated evaluation, or movement and affective modeling. Topic labels were assigned according to high-probability terms, representative documents, and semantic consistency within each topic. Accordingly, the topic labels are used as concise interpretive summaries of dominant word patterns, rather than as mutually exclusive research domains.

**Figure 8:**
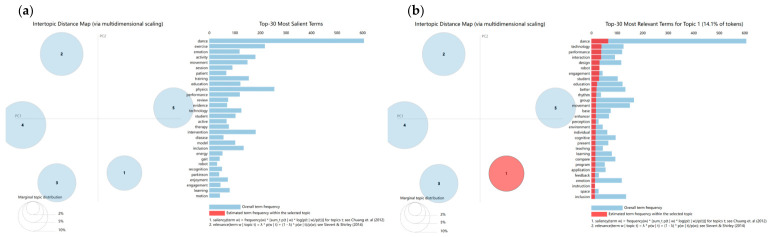

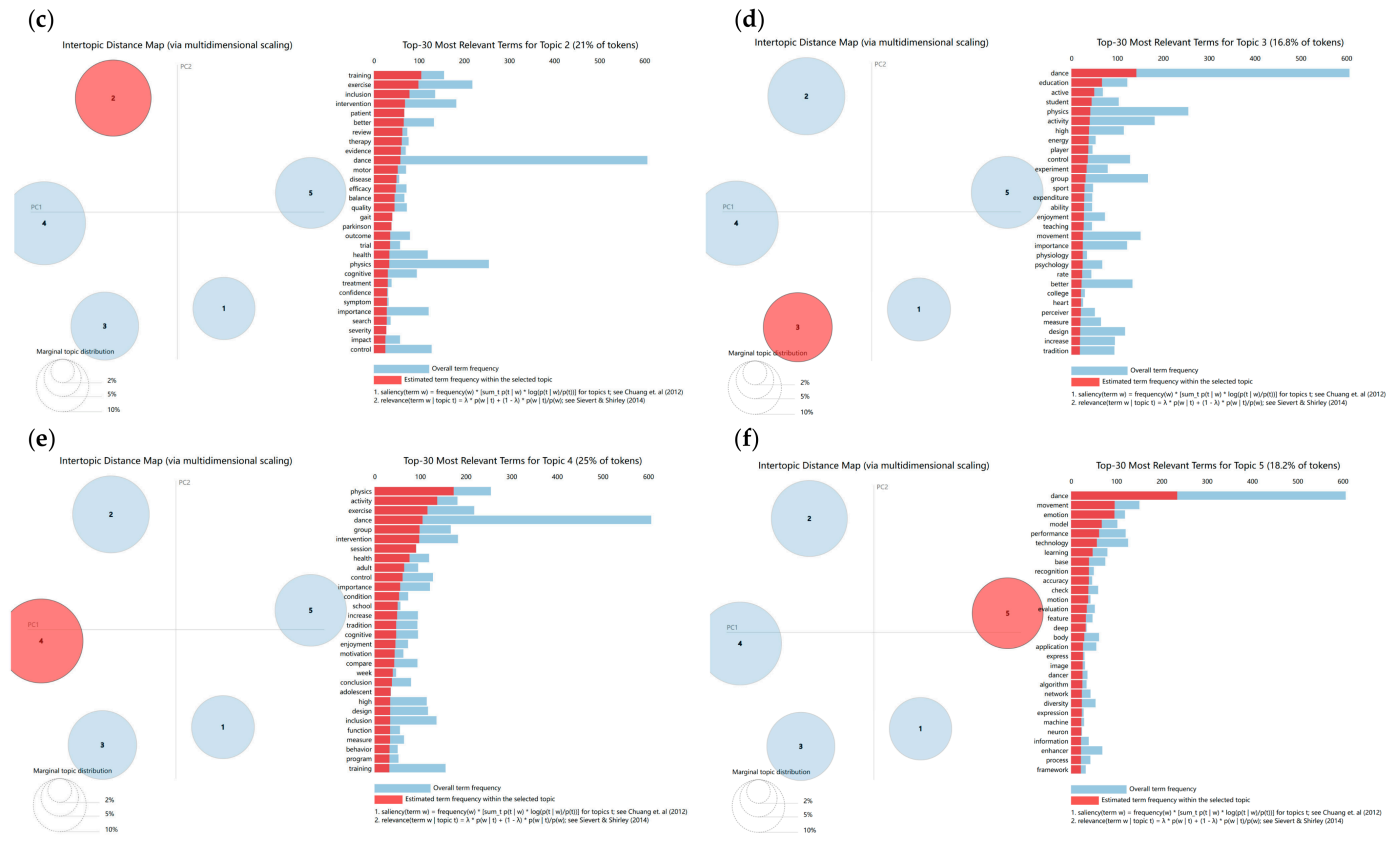
Topic Overview and analysis. (**a**) Analysis of the Five Topics; (**b**) Immersive interaction and intelligent instructional design; (**c**) Rehabilitation training and clinical evidence; (**d**) School-based exergaming and psychophysiological assessment; (**e**) Process management of physical-activity interventions and behavioral motivation; (**f**) Motion recognition and affective expression modeling. In the intertopic distance map, bubble size represents relative topic prevalence, and the distance between bubbles reflects semantic separation among topics. The accompanying bar charts show the most salient terms associated with each topic. Numbers in the bubbles indicate the five LDA topics, and colors are used to highlight the selected topic in each panel.

As shown in Figure 8a, the left panel displays the intertopic distance map obtained by multidimensional scaling, where each bubble represents one topic. The spatial distance between bubbles reflects semantic dissimilarity (greater distance corresponding to larger differences in word distributions), while bubble size indicates the marginal proportion of each topic in the corpus. In this map, Topics 2 and 1 have the largest bubbles, suggesting relatively high prevalence, whereas Topics 3–5 occupy smaller areas and represent more focused portions of the literature. The right panel presents the top-30 most salient terms: blue bars denote overall term frequencies in the entire corpus, and red bars indicate term frequencies estimated within the selected topic (relevance at λ = 1). High-salience terms such as dance, group, session, exercise, emotion and enjoyment provide an initial basis for interpreting the thematic content of each topic.

As shown in Figure 8b, Topic 1 is a large, application-oriented cluster (≈14.1% of tokens) centered on immersive dance interaction and technology-enhanced teaching. Its most relevant terms—dance, technology, performance, interaction, design, robot, engagement, student, education, rhythm, group, movement—are complemented by words such as perception, environment, individual, cognitive, teaching, learning, program, application, feedback, emotion, instruction, space and inclusion. Together, these terms indicate work on interactive dance or rhythm-based systems in which technical forms (e.g., robots, interactive installations) and instructional design are jointly used to shape students’ participation and learning outcomes. Studies in this topic typically treat dance or exergame activities not only as entertainment but as instructional media embedded in structured programs, with clear links between task design, feedback and educational objectives. At the same time, they examine learners’ engagement, emotional and cognitive responses, and individual differences within group settings, highlighting how immersive interactive environments can support intelligent, feedback-driven teaching in dance sport and related movement contexts [16,17]. This topic therefore reflects the education- and interaction-oriented part of the corpus, where digital or intelligent technologies are linked to dance/rhythm-related learning, engagement, and feedback.

As shown in Figure 8c, Topic 2 is another large, clinically oriented cluster (≈21% of tokens) that reflects typical structures of rehabilitation research. Its most relevant terms—training, exercise, intervention, patient, disease, treatment, therapy, outcome, trial, evidence, review, efficacy, quality—outline an intervention framework aimed at specific patient groups. Terms such as gait, balance, motor, symptom, and severity point to studies involving movement disorders and gait–balance impairments, where structured rehabilitation programs are used to improve functional performance and everyday mobility. The co-occurrence of trial, control, outcome, evidence, and review indicates a strong focus on randomized controlled trials and evidence synthesis, with emphasis on the effectiveness and replicability of interventions. Within this topic, dance appears alongside exercise and training, suggesting that dance or rhythm-based activities are treated as rehabilitation modalities rather than purely artistic content. The presence of cognitive and health further shows that functional and health-related outcomes serve as key evaluation endpoints. Overall, Topic 2 captures the strand of research on dance/exercise-based rehabilitation training and clinical evidence appraisal [18,19,20,21]. This topic reflects the rehabilitation-oriented part of the corpus, where dance or rhythm-based activity is examined alongside broader exercise and technology-supported training contexts.

As shown in Figure 8d, Topic 3 is a school-centered cluster (≈16.8% of tokens) focusing on physically active games and dance activities among students. Its most relevant terms—education, student, college, teaching, active, activity, sport, player, group, experiment, control—locate this topic in structured educational settings and typical school-based intervention or experimental designs. A second set of terms—energy, expenditure, rate, heart, physiology, psychology, measure, ability—highlights a strong emphasis on quantitative outcome indices, such as energy expenditure, heart-rate responses and physiological or psychological load. The occurrence of perceiver and related words suggests that some studies also incorporate perceived exertion or subjective ratings, forming a combined physiological–psychological evaluation framework. Together with dance and tradition, these patterns indicate that both exergames and traditional or folk dance are implemented in school curricula, with attention to instructional design and effectiveness. Overall, Topic 3 captures school-based movement and dance programs in which multiple physiological and psychological measures are used to assess “training-for-health” effects in student populations rather than clinical patients [22,23,24,25]. This topic has the clearest connection with exergaming and active video game research, where dance- or movement-based tasks are evaluated through both activity-related and psychological indicators.

As shown in Figure 8e, Topic 4 is a mid-sized cluster (≈25% of tokens) that, like Topic 3, centers on traditional physical activities and dance-based training, but with greater emphasis on the process of intervention delivery and the mechanisms of behavior change. Salient terms such as intervention, session, program, week point to curricula organized into structured blocks or multi-week training cycles, while behavior, motivation, enjoyment, inclusion highlight a focus on sustaining participation and motivational engagement rather than only verifying whether an intervention is “effective”. The co-occurrence of adult and adolescent indicates that both adults and youth are covered, typically in institutional settings such as school. Terms including function, cognitive, health, measure show that functional, cognitive and health-related indicators are collected alongside behavioural outcomes, and compare, control suggest the use of controlled designs to examine these relationships. Overall, Topic 4 can be characterised as work on “intervention project management and behavioural guidance”, stressing how the design of activities (design, condition) shapes adherence and participation effects (increase), and how these, in turn, link training programs to sustained behavioral change [5,20,26]. This topic captures the behavioral and program-delivery dimension of the corpus, with emphasis on motivation, enjoyment, inclusion, and participation across structured activity programs.

As shown in Figure 8f, Topic 5 is a computationally oriented cluster (≈18.2% of tokens) centered on modeling and evaluating dance and movement recognition. Salient terms such as recognition, accuracy, evaluation, check, model, algorithm, network, deep, neuron, machine, feature, and information indicate work that builds and tests machine-learning or deep-learning models, with explicit attention to model performance and validation procedures. In parallel, terms including dance, movement, motion, body, dancer, and image point to visual or sensor-based representations of dance movement and body dynamics as the primary inputs. The co-occurrence of emotion, express, and expression suggests that some studies extend beyond action classification to capture affective expression, performer style, or emotional content in dance sequences. Words such as framework, process, base, and application further imply systematic pipelines in which feature extraction, model training, and evaluation are organized into explicit frameworks before being applied to concrete tasks. Overall, Topic 5 represents research on AI- and VR-related motion and emotion recognition in dance contexts [27,28,29]. This topic represents the AI-oriented part of the corpus, where recognition, modeling, and automated evaluation are applied to dance movement, body representation, and affective expression.

#### 3.2.3. Topic Prevalence Distribution and Strength Analysis

**Figure 9:**
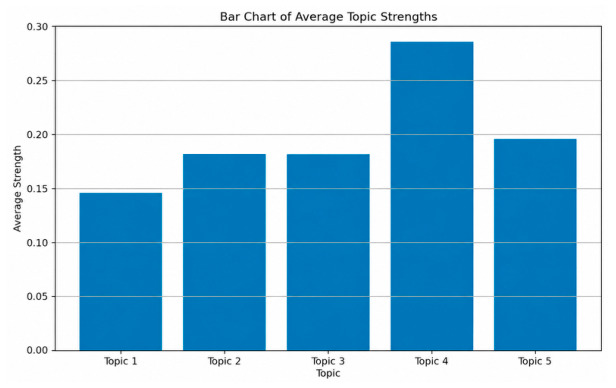
Bar chart of average strengths.

As shown in Figure 9, Topic 4 shows the highest strength (≈0.28), marking it as the most prominent research focus. A second tier is formed by Topics 5, 2 and 3 (≈0.20, ≈0.18 and ≈0.18, respectively), reflecting substantial but more evenly distributed attention to motion-recognition/affective modeling, rehabilitation training, and school-based psychophysiological assessment. Topic 1 has the lowest average strength (≈0.15), suggesting a comparatively smaller but still visible stream centred on immersive interaction and instructional design. Together, these values complement the marginal topic proportions and provide a quantitative basis for comparing the relative weight of the five thematic areas.

#### 3.2.4. Topic Interrelations and Multi-Topic Keyword Bridging

**Figure 10:**
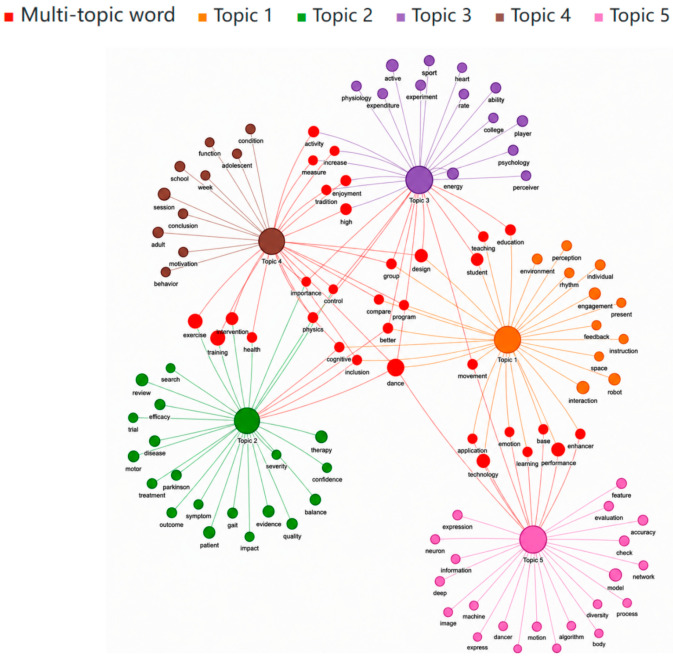
Topic–keyword network of intertopic relations and multi-topic bridging terms.

As illustrated in Figure 10, a topic–keyword network was constructed from the LDA results, where node size reflects term importance within its topic, node color indicates topic membership, and red nodes represent multi-topic words shared across at least two topics. These red nodes form the core semantic “spine” of the network. Terms such as dance and movement occupy central positions and link the intervention/technology stream (Topic 1), the process–behavior stream (Topic 4) and the clinical rehabilitation stream (Topic 2), showing how dance- or rhythm-based activities function as a common vehicle across educational, behavioral and clinical contexts. Another group of bridge terms—training, intervention, control, compare, group, measure, outcome—provides a shared methodological vocabulary for trial design and effect evaluation, enabling school-based trials, clinical rehabilitation and technology-enhanced programs to be discussed within a comparable evidence framework. A third cluster of red nodes—technology, application, program, learning, performance, emotion—anchors the link between system design and user-level outcomes, connecting Topic 1 (interactive teaching systems), Topic 5 (motion/affect recognition models) and Topic 3 (school-based psychophysiological assessment).

The overall structure of the network suggests that the five topics are clearly differentiated in semantic content yet connected through these bridge terms. Topic 2 aligns with clinical evidence and impairment-focused rehabilitation (trial, efficacy, gait, balance, Parkinson), Topic 4 emphasizes contextualized programs and behavioral change in everyday settings (school, adolescent, session, behavior), Topic 1 represents interactive teaching and engagement mechanisms (interaction, feedback, instruction, engagement, robot), Topic 3 highlights energy- and physiology-based measurement (energy, expenditure, physiology, heart rate), and Topic 5 clusters computational modeling and deep learning (algorithm, deep, network, recognition, accuracy). At the same time, the multi-topic words indicate that these strands are not isolated but converge on a shared research chain: “dance/movement-based intervention → program and behavioral design → physiological and psychological outcomes → AI-enabled motion and emotion modeling.”

#### 3.2.5. Topic Temporal Evolution Analysis

**Figure 11:**
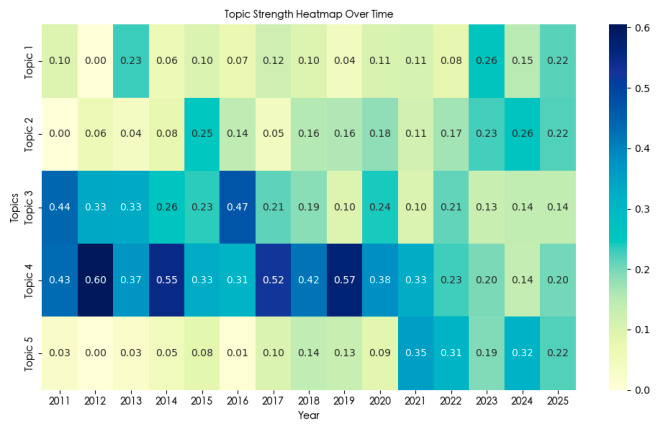
Topic Strength Heatmap Over Time (2011–2025).

**Figure 12:**
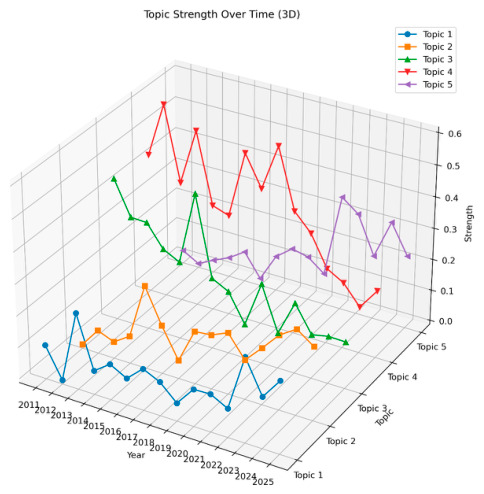
Topic Strength Over Time.

Figure 11 and Figure 12 present the year-by-year distribution of topic strengths from 2011 to 2025. Because the corpus contains 252 records and the number of records varies across years, these values should be interpreted as descriptive patterns of topic visibility within the WoSCC-indexed corpus rather than as statistically tested temporal trends. In the early years, Topics 3 and 4 showed comparatively higher values in several years, indicating that school-based exergaming, physical-activity programs, and behavioral or psychophysiological assessment were visible themes in the early part of the corpus. In more recent years, Topic 5 and Topic 2 became more visible in the annual topic-strength profiles, indicating greater representation within the retrieved records of AI-based motion or emotion recognition and rehabilitation-oriented dance or rhythm training. Topic 1 remained comparatively smaller but appeared consistently in recent years, reflecting the continued presence of immersive interaction and instructional design. Overall, the temporal figures are used to describe changes in the relative visibility of topics across publication years, not to establish statistically significant trends or causal shifts in research priorities.

## 4. Discussion

### 4.1. Overview of Main Findings and Contribution

This study examined AI-, VR-, and exergame-based dance and movement research on psychological outcomes from 2011 to 2025 by combining bibliometric analysis with LDA topic modeling. Based on 252 Web of Science records, co-occurrence and co-citation analyses were used to describe the structural features of the field, while the selected five-topic LDA solution was used to identify latent thematic patterns and their relative prominence over time. The results show that the literature is not organized around a single line of inquiry. Instead, it is distributed across five related areas: interactive teaching and engagement, rehabilitation training, school-based exergaming with psychophysiological assessment, process-focused behavioral programs, and AI-based motion and emotion recognition.

Across these areas, dance and dance-like exergames appear in educational, community, and clinical contexts. Psychological constructs such as enjoyment, motivation, self-efficacy, inclusion, and quality of life are often discussed alongside physical, functional, or performance-related outcomes [30,31,32,33,34]. The topic-bridging results also suggest lexical links among movement-based interventions, program design, psychophysiological and psychological measurement, and computational modeling of movement and affect. Compared with reviews that focus on exergames, VR rehabilitation, or AI recognition separately, this study provides a broader overview of how these lines of work appear within the same WoSCC-indexed research space. The main contribution of this study is to provide a structured overview of how AI-, VR-, and exergame-based dance and movement studies are connected to psychological outcomes. By clarifying the relationships among technology functions, movement contexts, and psychological constructs within the WoSCC-indexed corpus, the findings provide a basis for more targeted empirical studies and more precise outcome selection in future dance- and movement-based technology research.

Interpreting these findings through the three psychological outcome categories defined in the Introduction further clarifies the uneven psychological focus of this literature. Participation-related experiences were most visible in terms and topics related to exergaming, active video games, school-based activity, engagement, enjoyment, motivation, and exercise experience. Psychosocial functioning was reflected in terms such as self-efficacy, social interaction, inclusion, emotional expression, body representation, and quality of life, often in studies involving dance, rehabilitation, or technology-supported interaction. By contrast, mental health and well-being outcomes, including anxiety, depression, stress, loneliness, mood, and psychological well-being, were less consistently represented as central keyword or topic terms. This pattern suggests that psychological outcomes in this field have more often been addressed as participation-related or psychosocial indicators than as clearly defined mental-health targets. This distribution may partly reflect the disciplinary composition of the corpus. Studies grounded in education, sport, exercise, rehabilitation, or technology-design contexts tend to use psychological constructs to assess participation experience, intervention acceptability, or functional improvement, whereas mental-health outcomes usually require clearer psychological or clinical framing, validated instruments, and longer follow-up. Future studies should therefore define psychological targets more explicitly and use stronger theoretical integration when selecting outcome measures.

### 4.2. Thematic Insights: From Movement-Based Interventions to AI-Based Modeling

#### 4.2.1. Dance and Exergaming as Vehicles for Psychological Health

Across Topics 1, 3, and 4, dance and dance-like exergames appear mainly as structured movement activities in educational and everyday settings. School- and college-based studies combine active games, traditional or folk dance, and sport-related activities with measures such as energy expenditure, heart-rate response, perceived exertion, and psychological experience. Process-oriented studies also emphasize session structure, group formats, and multi-week programs. Terms such as motivation, enjoyment, inclusion, engagement, and group indicate that participation-related and social-experiential constructs are visible in this part of the literature [34,35,36]. The results suggest that dance and exergaming are commonly studied as engaging forms of physical activity, but the theoretical connection between short-term enjoyment, perceived competence, social interaction, and sustained participation remains unevenly developed. In this part of the literature, the technological contribution is mainly reflected in exergames, active video games, interactive feedback, and digitally structured learning environments, rather than in AI-based modeling alone.

#### 4.2.2. Clinical Rehabilitation and the Underexplored Mental-Health Dimension

Topic 2 reflects a clinically oriented strand in which dance or rhythm-based exercise is used as part of rehabilitation for neurological and movement disorders, especially Parkinson’s disease. Keywords such as gait, balance, symptom, severity, patient, trial, and evidence point to a strong focus on motor and functional outcomes in randomized trials and systematic reviews. Psychological variables appear mainly through broader indicators such as quality of life, fear of falling, depression, or anxiety, usually alongside motor performance rather than as the primary focus. This pattern suggests both strength and limitation. On the one hand, dance- or rhythm-based rehabilitation has been examined with relatively structured clinical designs. On the other hand, mental-health-related constructs are less visible than motor and functional terms in this topic. The current evidence base therefore appears to give more attention to functional improvement than to the psychological pathways through which rhythmic movement, social interaction, self-efficacy, or disease-related experience may influence well-being [37,38,39,40]. Here, VR and exergame technologies are most relevant when they function as structured rehabilitation environments, allowing rhythm-based movement tasks to be delivered with feedback, progression, and standardized monitoring. Future clinical studies could make this connection clearer by specifying psychological outcomes as primary or secondary targets and by linking them more explicitly to functional rehabilitation outcomes.

#### 4.2.3. AI-Driven Motion and Emotion Recognition as an Emerging Layer

Topic 5 adds a computational layer to the literature. Terms such as recognition, accuracy, algorithm, network, deep, and feature indicate studies that treat dance and movement as data sources for machine-learning or deep-learning models. Terms such as emotion, express, expression, dancer, and motion show that the objects of recognition include not only movement categories, but also affective or stylistic qualities. This topic is connected to the intervention-focused topics at the level of shared terms such as dance, movement, training, intervention, and emotion. However, this lexical overlap does not necessarily mean that AI-based recognition has been fully integrated into longitudinal intervention studies. In many cases, algorithm development relies on curated datasets or task-specific evaluations, while the present analysis cannot quantify the extent to which AI-based recognition has been linked to changes in mood, motivation, social functioning, or quality of life in real educational, community, or clinical programs [41,42,43,44]. Thus, the bridge-term analysis points to a potential connection between movement intervention, psychological measurement, and AI-based modeling, but further empirical work is needed to examine how this connection operates at the level of study design. Strengthening this link would help move AI-based recognition from technical classification toward feedback and assessment tools that can be embedded in VR, exergame, or other technology-supported dance and movement programs.

### 4.3. Practical Implications for Program and Technology Design

#### 4.3.1. Educational and Community Programs

The prominence of Topics 1, 3, and 4, together with terms such as motivation, enjoyment, inclusion, engagement, and group, suggests that psychological outcomes in educational and community settings are linked not only to the activity itself, but also to how dance and exergaming sessions are organized. These results support program designs that combine clear session structure, progressive task difficulty, peer interaction, and opportunities for feedback. In this context, psychological evaluation should not be limited to general satisfaction or enjoyment. It should be aligned with the intended program target, such as motivation, engagement, perceived competence, social connection, or inclusion. The repeated pairing of physiological and psychological terms also suggests the value of combined assessment. Objective indicators such as heart-rate response, energy expenditure, and activity intensity can be used together with validated measures of enjoyment, motivation, engagement, and self-efficacy. This type of assessment may help identify which formats are not only physically active, but also psychologically engaging, especially for students or community participants who may be less attracted to conventional sports.

#### 4.3.2. Rehabilitation Practice

In Topic 2, dance and rhythm-based exercise appeared in structured rehabilitation programs for neurological and movement disorders. Terms such as trial, evidence, gait, balance, symptom, and quality of life indicate that this strand has a strong functional and clinical orientation. For rehabilitation practice, the main implication is not simply to add psychological scales to existing protocols, but to define which psychological outcomes are expected to change and why. For example, studies aimed at improving daily functioning may include quality of life and fear of falling, while studies targeting psychosocial adaptation may include self-efficacy, mood, social participation, or perceived confidence in movement. Clearer reporting of dose, content, delivery mode, and technology format would also improve comparability across trials. This would make it easier to examine whether dance-, VR-, or exergame-based rehabilitation affects psychological outcomes directly, or mainly through improvements in mobility, balance, and participation.

#### 4.3.3. AI and VR System Development

Topic 5 highlights the technical strand of the corpus, in which dance and movement are modeled through recognition, accuracy, algorithm, network, deep learning, and emotion-related terms. This work shows that movement patterns, body dynamics, and affective expression can be represented computationally. However, these models are still more often developed for classification, recognition, or evaluation tasks than for testing psychological change in real training or rehabilitation settings. For system design, a practical next step is to connect AI-based recognition and feedback more directly with VR or exergame-based dance programs. For example, recognition models could support feedback on movement quality, engagement, synchrony, task difficulty, or emotional expression during teaching or rehabilitation sessions. However, systems that collect movement, behavioral, or affective data should be designed with clear safeguards. These include informed consent, data minimization, secure storage, transparent feedback rules, and careful consideration of bias or fairness in automated evaluation. In this sense, future AI/VR dance systems should not only improve recognition accuracy, but also show how recognition and feedback can support clearly defined psychological and behavioral outcomes.

### 4.4. Research Gaps and Future Directions

#### 4.4.1. Thematic Blind Spots in Psychological Outcomes

The keyword and topic results show that psychological outcomes are present in the corpus, but their visibility differs across constructs. Terms related to motivation, enjoyment, engagement, inclusion, and quality of life appeared more prominently in Topics 1, 3, and 4, especially in education-, exergaming-, and program-oriented studies [18,24,45,46]. By contrast, terms more directly related to anxiety, depression, stress, trauma-related problems, social withdrawal, or emotion regulation appeared less centrally in the keyword and topic results. This pattern suggests that the current literature is more strongly organized around participation-related and quality-of-life outcomes than around specific mental-health conditions or transdiagnostic psychological processes. Future research should therefore define psychological targets more explicitly. Studies should specify whether the intended outcome concerns motivation and engagement, psychosocial functioning, or mental health and well-being. This distinction is important because different targets require different theoretical assumptions, intervention designs, and measurement tools. For example, a study aimed at increasing enjoyment or engagement may require different design features from one aimed at reducing anxiety or improving social connectedness.

#### 4.4.2. Study Design, Follow-Up and Reporting

The topic structure suggests that different technological formats appear in different parts of the literature. Exergaming and active video games are more visible in student and physical-activity-oriented topics, VR/AR applications appear in teaching, rehabilitation, or immersive training contexts, and AI-based approaches are more visible in recognition and modeling topics [20,47,48,49]. Future intervention studies should therefore move beyond generic comparisons between “intervention” and “control” groups and examine which technology configurations add value for specific psychological targets. For example, multi-arm designs could compare conventional dance, VR-supported dance, exergame-based dance, and AI-feedback-supported dance. Within AI-feedback-supported dance, future studies could further compare visual movement correction, auditory rhythm cues, and haptic timing prompts to determine which feedback modes are most effective for improving outcomes such as self-efficacy, perceived competence, movement confidence, and continued participation. Other designs could compare fixed programs with adaptive task difficulty, human feedback with AI-assisted feedback, or individual immersive practice with socially interactive group formats. Follow-up assessments would also be useful, but they should be linked to technology-specific questions, such as whether AI-based feedback supports sustained engagement, whether VR environments increase perceived competence or social presence, or whether exergame formats improve adherence beyond the intervention period. In this way, study design improvements would be tied to the distinctive features of AI-, VR-, and exergame-based dance research rather than to generic trial recommendations.

#### 4.4.3. Measurement Quality and Theoretical Integration

The present bibliometric and topic-modeling analysis cannot directly evaluate the psychometric quality of measures used in individual studies. However, the keyword and topic results suggest that measurement-specific terminology and named psychological instruments were not prominent thematic anchors in the corpus. This does not mean that validated scales were absent, but it indicates that measurement transparency and construct alignment deserve more attention in future work. Future studies should report psychological instruments more explicitly and align them with the intended outcome domain. Studies focused on motivation and engagement should use validated measures of enjoyment, intrinsic motivation, flow, perceived competence, or engagement. Studies focused on psychosocial functioning should consider self-efficacy, body image, social connectedness, inclusion, or emotional expression. Studies focused on mental health and well-being should use appropriate measures of anxiety, depression, stress, quality of life, or psychological well-being. Theoretical frameworks such as self-determination theory, social-cognitive models, embodied cognition, and emotion-regulation theory may be useful, but they should be linked to specific constructs and hypotheses rather than cited only as broad background.

#### 4.4.4. Connecting AI-Based Modeling with Real-World Interventions

Topic 5 and the bridge-term analysis point to a potentially important connection between computational modeling and intervention-oriented research. Terms such as dance, movement, emotion, training, and intervention connect AI-based recognition with other parts of the corpus. However, these links appear mainly at the level of shared vocabulary. They do not necessarily show that AI-based recognition has been integrated into longitudinal intervention designs that evaluate psychological change [50,51]. Future research could address this gap by collecting kinematic, physiological, and affective signals alongside validated psychological measures in real educational, community, and clinical programs. AI-based recognition modules could then be tested not only for classification accuracy, but also for their ability to support feedback, adaptation, engagement, and psychological assessment within VR dance or exergame platforms. This would help connect technical model development with intervention evaluation by clarifying how AI-supported feedback, adaptation, or assessment can contribute to psychological outcomes in dance and movement contexts.

#### 4.4.5. Diversity of Settings and Populations

The country, institution, and topic distributions suggest that the current WoSCC-indexed corpus is concentrated in a limited set of regions, institutions, and populations. Children, adolescents, students, and specific clinical groups are more visible in the topic structure, whereas older adults without neurological diagnoses, low- and middle-income settings, and culturally diverse forms of dance, dance sport, and community dance appear less visible. Future research should therefore test whether AI-, VR-, and exergame-based dance and movement programs are feasible, acceptable, and psychologically meaningful across a wider range of populations and cultural contexts. Comparative studies across age groups, dance forms, delivery formats, and resource settings could clarify when technology-supported dance adds value beyond conventional movement programs and when simpler, low-technology formats may be more appropriate.

### 4.5. Strengths and Limitations

This study adopts an integrated methodological approach that combines bibliometric mapping with LDA-based topic modeling and topic-strength/temporal analyses. This approach enables a multi-level examination of AI-, VR-, and exergame-based dance and movement research on psychological outcomes by linking structural patterns, thematic clusters, and changes in research priorities over time.

Several limitations should also be noted. First, the study was restricted to English-language records indexed in the Web of Science Core Collection. WoSCC was selected to preserve data stability and ensure consistency across bibliometric and topic-modeling analyses. However, relevant publications indexed in other databases, such as PubMed or Scopus, or published in languages other than English may have been excluded. Therefore, the findings should be interpreted as a mapping of the WoSCC-indexed English-language literature rather than as an exhaustive cross-database review. Future research could incorporate multiple databases and languages to test the robustness of the identified topic structure.

Second, bibliometric and topic-modeling methods describe patterns in the literature, but they do not evaluate intervention effectiveness or the quality of individual studies. Co-occurrence patterns, topic proportions, and topic-strength curves should therefore be interpreted as descriptive indicators of research visibility and thematic organization, not as evidence of causal effects or clinical efficacy. Temporal topic-strength patterns may also be influenced by annual publication volume, journal coverage, funding directions, database indexing, and the composition of the retrieved corpus, and therefore should not be interpreted as direct evidence of changes in underlying research priorities. In addition, the LDA analysis was based on terms appearing in titles and abstracts and could not distinguish primary outcomes, secondary outcomes, validated scale-based measures, or descriptive contextual mentions. Therefore, psychological terms in the topic-modeling results should be interpreted as textual representations within the corpus rather than as evidence of formally measured outcome variables.

Third, the LDA results depend on the selected corpus, preprocessing decisions, topic number, and model settings. Although the K = 5 solution provided an interpretable structure based on coherence, perplexity, and topic interpretability, other topic solutions may emphasize different levels of thematic detail. The topic labels should therefore be understood as concise interpretations of probabilistic patterns rather than fixed categories of research.

## 5. Conclusions

This study mapped WoSCC-indexed research on AI-, VR-, and exergame-based dance and movement studies related to psychological outcomes from 2011 to 2025. The findings show that this literature is organized around several connected themes, including school-based exergaming and psychophysiological assessment, rehabilitation-oriented dance or rhythm training, immersive interaction and technology-supported teaching, behavioral program design, and AI-based motion or emotion recognition. These themes should not be read as separate research domains, but instead as related strands within a continuous research space where technology functions, movement contexts, and psychological constructs are unevenly connected.

The findings also point to several areas that require more focused empirical work. AI-based motion or emotion recognition needs to be linked more directly with longitudinal psychological outcomes, including anxiety, depression, stress, well-being, self-efficacy, and sustained participation. Comparative studies are also needed to examine the added value of different technology layers, such as conventional dance, VR-supported dance, exergame-based dance, and AI-feedback-supported dance, within the same population. Future intervention studies should define psychological targets more clearly and use validated measures, especially when psychological outcomes are examined alongside functional, training-related, or technical outcomes. Overall, future research should move toward more targeted and longitudinal designs that examine how specific AI-, VR-, or exergame-based features influence movement skills, exercise experience, participation, and psychological outcomes in dance and movement contexts.

## Figures and Tables

**Table 1:** The top five references by co-citation frequency.

Ranking	Cited Reference	Year	Frequency	Centrality
1	Active video games to promote physical activity in children and youth: a systematic review	2010	12	0.07
2	Children’s physical activity levels and psychological correlates in interactive dance versus aerobic dance	2013	11	0
3	Impact of exergaming on young children’s school day energy expenditure and moderate-to-vigorous physical activity levels	2017	11	0.05
4	Twelve weeks of dance exergaming in overweight and obese adolescent girls: Transfer effects on physical activity, screen time, and self-efficacy	2017	11	0.03
5	A meta-analysis of active video games on health outcomes among children and adolescents	2015	9	0.08

Note: Co-citation frequency refers to the number of co-citations within the analyzed WoSCC corpus. Betweenness centrality reflects the extent to which a reference connects different co-citation clusters; therefore, a reference may have a high co-citation frequency but a low centrality value.

## Data Availability

No new data were created or analyzed in this study.

## Supplementary Materials

The following supporting information can be downloaded at: https://www.mdpi.com/article/10.3390/healthcare14121662/s1, Table S1: Top 10 terms with weights for each LDA topic.

## Abbreviations

The following abbreviations are used in this manuscript:

AI	Artificial Intelligence
VR	Virtual Reality
LDA	Latent Dirichlet Allocation
WoS	Web of Science
LLR	Log-likelihood ratio
C_v	Coherence score
