# Supplementary material for: AI-, VR-, and Exergame-Based Dance and Movement Research on Psychological Outcomes: A Bibliometric and Topic-Modeling Analysis of Thematic Structure and Development
Source: Healthcare (Basel). 2026 Jun 11;14(12):1662. doi: 10.3390/healthcare14121662 (PMC13299272; doi:10.3390/healthcare14121662)
Supplement: Supplementary file 1 [file healthcare-14-01662-s001.zip › healthcare-4334538-supplementary.pdf]

**Supplementary Table S1. Top 10 terms with weights for each LDA topic**

| Topic | Label | Top 10 terms with weights for each LDA topic |
|---|---|---|
| Topic 1 | Immersive interaction and intelligent instructional design | dance(0.074), technology(0.044), performance(0.043), interaction(0.043), design(0.036), robot(0.035), engagement(0.034), student(0.034), education(0.025), better(0.022). |
| Topic 2 | Rehabilitation training and clinical evidence | training(0.057), exercise(0.053), inclusion(0.043), intervention(0.037), patient(0.036), better(0.036), review(0.034), therapy(0.033), evidence(0.032), dance(0.032). |
| Topic 3 | School-based exergaming and psychophysiological assessment | dance(0.109), education(0.051), active(0.038), student(0.034), physics(0.032), activity(0.031), high(0.030), energy(0.029), player(0.028), control(0.027). |
| Topic 4 | Process management of physical-activity interventions and behavioral motivation | physics(0.073), activity(0.058), exercise(0.049), dance(0.044), group(0.041), intervention(0.041), session(0.038), health(0.032), adult(0.027), control(0.026). |
| Topic 5 | Motion recognition and affective expression modeling | dance(0.140), movement(0.058), emotion(0.057), model(0.041), performance(0.037), technology(0.034), learning(0.029), base(0.024), recognition(0.024), accuracy(0.024). |
